# Supplementary material for: Vertical transmission of Dengue virus type-3 and metagenomic virome profiles of Aedes aegypti mosquitoes collected in Kisumu, Kenya
Source: PLoS One. 2025 Jul 2;20(7):e0315492. doi: 10.1371/journal.pone.0315492 (PMC12221173; doi:10.1371/journal.pone.0315492)
Supplement: Attachment — Submitted filename: PlosOne commentaires 30 12 2024.docx [file pone.0315492.s001.docx]

The authors report in their article the vertical transmission of dengue virus and other insect specific viruses in *Aedes aegypti* collected in Kisumu, Kenya. The use of cell culture for virus isolation and NGS technique to detect the virome were interesting as approach but it remains some gaps to fulfil.

Abstract

Line 34: change “processed for” by “analysed using”

Line 78-85: please add the number of dengue cases

Line 86: you have to write complete name before using abbreviation Insect Specific Viruses (ISVs) ;

Line 134-135: repetition of the idea, please delete it.

Line 169-170 : “Larvae and pupae were collected” is better

Line 174 : the samples were georeferenced

Line 194: adults were completely crushed or a part of the them?

Comparison between pools and superpools

Line 257; which model have you used for your calculation.

Line 261: move weather data to material & methods chapter where you describe the study area

Line 271-276: reformulate, detail your results and give significant of the abbreviation in the table 1

Results

Miss of the table containing mosquito species identification

Line 281: change the title from dengue to viral isolation (follow your M&M plan) and give more details about your results: day of CPE, …

Table 2 coverage rate is not reported which is important also.

Have you amplified NS5 protein from the isolate? It is not reported in M&M for dengue classification? And what is its bootstraps value? What is the threshold used in the tree for nodes value bootstrap?

Why did you not perform a second qRT-PCR or end point Rt-PCR to confirm you dengue virus? Because 726bp is not enough and represent only 7% of the total genome

Line 328: unclassified virus formed a clade with Role-bu virus

In table 2 only the result of three superpools are shown , check it again

Figure 5, accession number do not correspond to any data neither in Uniprot nor in GenBank databases, please can you explain?

From my point of view, % of resemblance corresponding to 79% is to low to say that the identified viruses belong to the same family and using RdRp gene of the corresponding viruses can give faults results.

Low quality of discussion, there are less than 10 references in the discussion and it is more a narration then discussion and comparison of results with other studies. The authors took the results and re-presented them.

General comments on NGS approach:

While the approach is certainly comprehensive and relies upon appropriate tools for the metagenomic sequencing and subsequent bioinformatic analysis, it can certainly benefit from a review of the following:

-There is a distinct lack of external validation of the results obtained through the CZ ID platform. The study could be improved if a secondary command-line-based pipeline or alternative tools were employed to ensure the confirmation, and more importantly reproducibility of the results.

-A potential pitfall with the approach is in leveraging cell culture and passaging samples in cellular systems and media, which can potentially risk the introduction of artificial diversity in the sample and hence may hinder the accurate capture of the original viral diversity and can lead to skewed representations.

-Justifications are lacking as it pertains to the choice of thresholds, namely in the size of the contigs, the statistical e-value threshold, and read depth. While the e-value threshold is quite stringent in database searches and helps avoid spurious results and false positives, a tradeoff exists with the identification of novel viruses that may share minimal similarities with the entries of the chosen database and are hence lost as false negatives. In a similar vein, the application of a read depth threshold of ≥10 may cause loss of potentially biologically relevant viruses to be detected if they exist in lower abundance or are fragmented.

-The Viral Identification step relies exclusively on the comprehensiveness of the NCBI’s viral database that although extensive, remains rapidly evolving with continuous new entries added to the database. It may prove useful to likewise perform a secondary search against more curated databases to improve the detection of novel viruses and provide a more accurate representation of the virome. The secondary confirmation, as noted above is also important for ensuring confirmation and reproducibility of the results.

-The inclusion of controls is imperative to the detection of contaminations that can often result during sequencing and is an integral component for verification of the steps throughout the experimental procedure.
