# Supplementary material for: Vertical transmission of Dengue virus type-3 and metagenomic virome profiles of Aedes aegypti mosquitoes collected in Kisumu, Kenya
Source: PLoS One. 2025 Jul 2;20(7):e0315492. doi: 10.1371/journal.pone.0315492 (PMC12221173; doi:10.1371/journal.pone.0315492)
Supplement: Attachment — Submitted filename: Comment for MS- PONE-D-24-54015.docx [file pone.0315492.s002.docx]

General comment

Your article is very good; however, I have few minor comments that could improve the quality and readability of your article.

Major comment/General Question

1) Your sampled house is to small and it is difficult to calculate entomological indices - it is better if you remove this part.
2) Why your choses only dengue type 3 viruses? Do you have any justification why you focus on type 3 viruses?
3) Is one-month weather data enough to relate with Aedes mosquito’s density.
4) Line 345 – 355: “…WHO thresholds for dengue outbreak risk; CI >1%, HI > 1% and BI > 5 …” is this statement true? See the following link: <https://iris.who.int/bitstream/handle/10665/218621/WER4649_493-500.PDF> , <https://tropmedhealth.biomedcentral.com/articles/10.1186/s41182-020-00207-w>
5) What type of adult mosquito collection method used? or you collect only immature stage?

Minor Comments:

Background

1. Line 54: “There are two type of Aedes…” I think such type of classification is not common for Aedes. I try two see your reference paper and I couldn’t find such type of classification. I think it is important, if you paraphrase it.
2. Line 106: …. “(from one host to another)” …. What you mean by another? Is it host or mosquito? … human-mosquito-human – this is horizontal transmission.

Result

1. Line 272, “… 5 houses sampled in the present study, 3 were positive for immature mosquitoes” this sample size too small. See on line 184, minimum sampled houses required to calculate Entomological indices.
2. Line 278: Table 1, remove rank columns

References

1. Use endnote or any other reference software,
2. Line 69,72,76….. Correct your citation with in the document – for example “(10)(9)” (10,9).
3. Reference 31 (line 630) “Golding MAJ, Noble SAA, Khouri NK, Yarde RNAL, Ali I, Sandiford SL. Natural vertical transmission of dengue virus in Latin America and the Caribbean : highlighting its detection limitations and potential significance. Parasit Vectors [Internet]. 2023;1–13. Available from: <https://doi.org/10.1186/s13071-023-06043-1>”

The correct reference: Golding, M.A., Noble, S.A., Khouri, N.K., Layne-Yarde, R.N., Ali, I. and Sandiford, S.L., 2023. Natural vertical transmission of dengue virus in Latin America and the Caribbean: highlighting its detection limitations and potential significance. *Parasites & Vectors*, *16*(1), p.442.
