# Supplementary material for: Vertical transmission of Dengue virus type-3 and metagenomic virome profiles of Aedes aegypti mosquitoes collected in Kisumu, Kenya
Source: PLoS One. 2025 Jul 2;20(7):e0315492. doi: 10.1371/journal.pone.0315492 (PMC12221173; doi:10.1371/journal.pone.0315492)
Supplement: Attachment — Submitted filename: Responses to reviewers.docx [file pone.0315492.s004.docx]

**Responses to reviewers**

The authors report in their article the vertical transmission of dengue virus and other insect specific viruses in *Aedes aegypti* collected in Kisumu, Kenya. The use of cell culture for virus isolation and NGS technique to detect the virome were interesting as approach but it remains some gaps to fulfil.

Abstract

Line 34: change “processed for” by “analysed using”

**Response: Line 38 this has been addressed to ‘analysed using’**

Line 78-85: please add the number of dengue cases

**Response: Line 79-89 this has been addressed and the number of dengue cases have been added**

Line 86: you have to write complete name before using abbreviation Insect Specific Viruses (ISVs);

**Response: Line 90 complete name has been added**

Line 134-135: repetition of the idea, please delete it.

**Response: Line 137-139. This has been deleted**

Line 169-170: “Larvae and pupae were collected” is better

**Response: Line 192-193. This has been corrected**

Line 174: the samples were georeferenced

**Response: Line 197: This has been addressed**

Line 194: adults were completely crushed or a part of the them?

**Response: Line 220: yes completely crushed**

Comparison between pools and superpools

**Response: Line 228-230.**

Line 257; which model have you used for your calculation.

**Response: Line 287-288. GTR+G4 (General Time Reversible + Gamma)**

Line 261: move weather data to material & methods chapter where you describe the study area

**Response: Line 180-190. This has moved to methods**

Line 271-276: reformulate, detail your results and give significant of the abbreviation in the table 1

**Response: Line 296-299.This has been addressed**

Results

Miss of the table containing mosquito species identification

**Response: The table is missing because the morphological identification had already been done by the entomologists and determined to be *Aedes aegypti* species which was then processed for analysis.**

Line 281: change the title from dengue to viral isolation (follow your M&M plan) and give more details about your results: day of CPE, …

**Response: Line 304. This has been addressed**

Table 2 coverage rate is not reported which is important also.

**Response: Line 327-328 Query cover has been added on the last column to show the coverage rate**

Have you amplified NS5 protein from the isolate? It is not reported in M&M for dengue classification? And what is its bootstraps value? What is the threshold used in the tree for nodes value bootstrap?

**Response:**

- **Line 251-254, 306: PCR section has been added in methodology and results**
- **Fig 3B. bootstrap value is 100 and the threshold for tree nodes is 70**

Why did you not perform a second qRT-PCR or end point Rt-PCR to confirm you dengue virus? Because 726bp is not enough and represent only 7% of the total genome

**Response**

- **As stated above we performed qRT-PCR prior to sequencing.**
- **Line 405- 407: The concern of the reviewer has been addressed by the additional information in the manuscript**

Line 328: unclassified virus formed a clade with Role-bu virus

**Response: Line 355 this has been addressed as mentioned**

In table 2 only the result of three superpools are shown, check it again

**Response: Superpool 2 is missing because after blast analysis on the contigs obtained, there was no significant hit, thus it was not included.**

Figure 5, accession number do not correspond to any data neither in Uniprot nor in GenBank databases, please can you explain?

**Response: The sequences were deposited in GenBank in April 2024 but had not been made public at the time of manuscript submission. They became public February 2025, now they are accessible.**

From my point of view, % of resemblance corresponding to 79% is to low to say that the identified viruses belong to the same family and using RdRp gene of the corresponding viruses can give faults results.

**Response**

- **Table 2. The BLAST identity of 79% indicate that our sequence is closely related but not identical to the hit.**
- **Fig 5C. Strong bootstrap support (89%) in the phylogenetic analysis shows that the detected virus represents a distinct species or a highly divergent strain within the Iflaviridae family.**
- **The RNA-dependent RNA polymerase (RdRp) region was selected for phylogenetic analysis because it retains essential functional motifs that facilitate robust sequence alignment and accurate tree construction. Additionally, the gene has been widely used by researchers who are doing similar work.**
- [**https://pmc.ncbi.nlm.nih.gov/articles/PMC4475995/pdf/pnas.201502036.pdf**](https://pmc.ncbi.nlm.nih.gov/articles/PMC4475995/pdf/pnas.201502036.pdf)

Low quality of discussion, there are less than 10 references in the discussion and it is more a narration then discussion and comparison of results with other studies. The authors took the results and re-presented them.

**Response**

- **More details have been added in the discussion section to improve the quality.**

General comments on NGS approach:

While the approach is certainly comprehensive and relies upon appropriate tools for the metagenomic sequencing and subsequent bioinformatics analysis, it can certainly benefit from a review of the following:

-There is a distinct lack of external validation of the results obtained through the CZ ID platform. The study could be improved if a secondary command-line-based pipeline or alternative tools were employed to ensure the confirmation, and more importantly reproducibility of the results.

**Response**

- **Line 265-268. This has been addressed**

-A potential pitfall with the approach is in leveraging cell culture and passaging samples in cellular systems and media, which can potentially risk the introduction of artificial diversity in the sample and hence may hinder the accurate capture of the original viral diversity and can lead to skewed representations.

- **Response Line 244 addresses this.**
- **Thank you for your insightful comment. We recognize that using cell culture and passaging samples in cellular systems can introduce potential biases and affect the viral diversity due to the selective pressures of the culture environment. To minimize the introduction of artificial diversity, we restricted ourselves to a passage of not more than 2.**

-Justifications are lacking as it pertains to the choice of thresholds, namely in the size of the contigs, the statistical e-value threshold, and read depth. While the e-value threshold is quite stringent in database searches and helps avoid spurious results and false positives, a tradeoff exists with the identification of novel viruses that may share minimal similarities with the entries of the chosen database and are hence lost as false negatives. In a similar vein, the application of a read depth threshold of ≥10 may cause loss of potentially biologically relevant viruses to be detected if they exist in lower abundance or are fragmented.

**Response: Line 270-272**

- **Regarding the contig size, we selected a size threshold that balanced between sensitivity and accuracy in assembling complete viral genomes, ensuring that we captured large enough fragments to confidently identify viral species while minimizing the inclusion of short, potentially erroneous sequences. The read depth of ≥10 was selected to ensure that the detected sequences were robust enough for reliable downstream analysis.**
- **In terms of the e-value threshold, we adopted a stringent value to reduce the risk of false positives in database searches. As you correctly pointed out, this may result in the exclusion of novel viruses that share minimal sequence similarity with known viral entries. However, the decision was made to prioritize high confidence in our results and to avoid the inclusion of spurious matches. We agree that this can lead to the potential loss of novel viruses, and we plan to further explore this limitation in future studies by applying less stringent e-value thresholds or utilizing broader databases, such as custom viral reference sets, to capture a wider range of viral diversity.**

-The Viral Identification step relies exclusively on the comprehensiveness of the NCBI’s viral database that although extensive, remains rapidly evolving with continuous new entries added to the database. It may prove useful to likewise perform a secondary search against more curated databases to improve the detection of novel viruses and provide a more accurate representation of the virome. The secondary confirmation, as noted above is also important for ensuring confirmation and reproducibility of the results.

**Response Line 273-276**

- **Thank you for your valuable feedback. We acknowledge that relying solely on the NCBI viral database may limit the detection of novel or underrepresented viruses, given the continuous expansion of viral sequence entries. However, to enhance the accuracy of our viral identification, we performed a secondary confirmation using non-redundant protein database (nr). This additional step helped validate our results and confirm the viral sequences identified in our study.**

-The inclusion of controls is imperative to the detection of contaminations that can often result during sequencing and is an integral component for verification of the steps throughout the experimental procedure.

**Response: Line 259-260. This has been addressed in the manuscript**

General comment

Your article is very good; however, I have few minor comments that could improve the quality and readability of your article.

Major comment/General Question

1) Your sampled house is to small and it is difficult to calculate entomological indices - it is better if you remove this part.

**Response**

- **Thank you for your feedback. While we acknowledge that the sampled area is not large, it is a car garage with 5 houses, that provided multiple breeding sites for mosquitoes. Importantly, we included this site because there was a suspected dengue case (Unpublished) from a nearby hospital, making it a relevant location for vector surveillance. Given the potential epidemiological significance, we believe it is important to retain this section to provide context on possible mosquito breeding habitats in areas linked to dengue cases.**

2) Why your choses only dengue type 3 viruses? Do you have any justification why you focus on type 3 viruses?

**Response**

- **Thank you for your question. Our study did not selectively focus on Dengue virus type 3 (DENV-3); rather, we conducted unbiased virus surveillance in mosquitoes, and DENV-3 was the serotype detected in our samples. The identification of DENV-3 was based on sequencing results, not preselection. The presence of DENV-3 in our surveillance samples is epidemiologically significant, as it suggests active circulation within the mosquito population, making our findings relevant for public health monitoring and outbreak preparedness.**

3) Is one-month weather data enough to relate with Aedes mosquito’s density.

**Response**

- **We acknowledge that mosquito population dynamics are influenced by long-term climatic trends, and ideally, extended weather data would provide a more comprehensive analysis. However, our study aimed to capture short-term fluctuations in Aedes mosquito density in relation to immediate environmental conditions, which is why we used one-month weather data. Previous studies have shown that temperature, rainfall, and humidity can influence mosquito breeding and activity within short timeframes, particularly in urban settings where container-breeding Aedes mosquitoes respond rapidly to environmental changes. While longer-term data would strengthen the analysis, the one-month period still provides valuable insights into how weather conditions may have influenced mosquito densities during the study period.**

4) Line 345 – 355: “…WHO thresholds for dengue outbreak risk; CI >1%, HI > 1% and BI > 5 …” is this statement true? See the following link: <https://iris.who.int/bitstream/handle/10665/218621/WER4649_493-500.PDF> , <https://tropmedhealth.biomedcentral.com/articles/10.1186/s41182-020-00207-w>

- **Thank you for your observation. We recognize that different classification systems exist for interpreting entomological indices in dengue surveillance. Our study initially referenced the commonly used thresholds (CI >1%, HI >1%, BI >5%), but we acknowledge that the alternative classification cited by the reviewer provides a more detailed risk stratification. Our classification has been used in similar work as shown by:**
  - **Lutomiah et al. (2016), which discusses entomological investigations during the 2013–2014 dengue outbreak in Mombasa, Kenya.**
- **With our observed values (HI: 60, CI: 57, BI: 660), the entomological risk remains extremely high under both classification schemes. Specifically:**
  - **HI (60%) exceeds the high-risk threshold (>35%),**
  - **CI (57%) is well above the high-risk threshold (>20%),**
  - **BI (660) far surpasses the high-risk threshold (>50).**
- **Given these values, our findings strongly indicate a heightened risk of dengue transmission if the virus is present and human susceptibility is high.**

5) What type of adult mosquito collection method used? or you collect only immature stage?

**Response**

- **Thank you for your question. In our study, we primarily focused on immature mosquito collection (larvae and pupae) from breeding habitats to assess Aedes mosquito densities and virus presence. The larvae and pupae were collected from various water-holding containers, such as tires and jerrycans, and reared to adulthood for species identification and virus screening.**

Minor Comments:

Background

1. Line 54: “There are two type of Aedes…” I think such type of classification is not common for Aedes. I try two see your reference paper and I couldn’t find such type of classification. I think it is important, if you paraphrase it.

**Response: Line 55: this has been paraphrased**

2. Line 106: …. “(from one host to another)” …. What you mean by another? Is it host or mosquito? … human-mosquito-human – this is horizontal transmission.

**Response: Line 111: This has been addressed to ‘human-mosquito-human cycle’**

Result

1. Line 272, “… 5 houses sampled in the present study, 3 were positive for immature mosquitoes” this sample size too small. See on line 184, minimum sampled houses required to calculate Entomological indices.

**Response**

- **Thank you for your feedback. We acknowledge that the sample size of 5 houses may be considered small, particularly when calculating entomological indices, which typically require larger sample sizes to ensure statistical reliability. However, our study aimed to provide preliminary surveillance data from this geographic area, and these 5 houses were the only standing structures. We based this study on their potential relevance to dengue transmission, as indicated by local health reports and previous dengue cases. While we agree that a larger sample size would strengthen the analysis of entomological indices, the focus of this study was on virus detection from field-collected mosquito samples rather than comprehensive vector abundance estimation.**

2. Line 278: Table 1, remove rank columns
   - **Response: Line 302: This has been addressed**

References

1. Use endnote or any other reference software,

**Response**

- **We use Mendeley Desktop for reference management in our work, we have ensured that all citations in the manuscript are properly formatted according to the journal's guidelines.**

2. Line 69,72,76….. Correct your citation with in the document – for example “(10)(9)” (10,9).

**Response: Line 70, 73, 76. This has been addressed**

Reference 31 (line 630) “Golding MAJ, Noble SAA, Khouri NK, Yarde RNAL, Ali I, Sandiford SL. Natural vertical transmission of dengue virus in Latin America and the Caribbean : highlighting its detection limitations and potential significance. Parasit Vectors [Internet]. 2023;1–13. Available from: <https://doi.org/10.1186/s13071-023-06043-1>”

The correct reference: Golding, M.A., Noble, S.A., Khouri, N.K., Layne-Yarde, R.N., Ali, I. and Sandiford, S.L., 2023. Natural vertical transmission of dengue virus in Latin America and the Caribbean: highlighting its detection limitations and potential significance. *Parasites & Vectors*, *16*(1), p.442.

**Response:**

**Line 667: This has been addressed in the document.**

**Revision 1**

When submitting your revision, we need you to address these additional requirements.

1.Please ensure that your manuscript meets PLOS ONE's style requirements, including those for file naming. The PLOS ONE style templates can be found at

<https://journals.plos.org/plosone/s/file?id=wjVg/PLOSOne_formatting_sample_main_body.pdf> and

[https://journals.plos.org/plosone/s/file?id=ba62/PLOSOne_formatting_sample_title_authors_affiliations.pdf](https://track.editorialmanager.com/CL0/https:%2F%2Fjournals.plos.org%2Fplosone%2Fs%2Ffile%3Fid=ba62%2FPLOSOne_formatting_sample_title_authors_affiliations.pdf/1/010f0195cb2ffe30-355d8bf0-d2ba-4f68-a0fc-96da6cad24db-000000/XDWB2OweJTQSmeLiLuHeLvsZPEscWTYaQ4J9oLwj71I=203)

**Response 1: The manuscript has been formatted according to PLOS ONE’s styling requirements.**

2. In your Methods section, please provide additional information regarding the permits you obtained for the work. Please ensure you have included the full name of the authority that approved the field site access and, if no permits were required, a brief statement explaining why.

**Response 2: Ethical approval was obtained from the Kenya Medical Research Institute (KEMRI) Scientific and Ethics Review Unit (SERU) under protocol number KEMRI/SERU/CCR/4702 and WRAIR# 3101. Permission to conduct the study was granted by the National Council for Science, Technology, and Innovation (NACOSTI).**

3. Please note that funding information should not appear in any section or other areas of your manuscript. We will only publish funding information present in the Funding Statement section of the online submission form. Please remove any funding-related text from the manuscript.

**Response 3: Funding information has been removed from the manuscript**

4. We note that the grant information you provided in the ‘Funding Information’ and ‘Financial Disclosure’ sections do not match.

When you resubmit, please ensure that you provide the correct grant numbers for the awards you received for your study in the ‘Funding Information’ section.

**Response 4: This are the correct grant numbers. ‘This work was funded by the Armed Forces Health Surveillance Branch (AFHSB) and its Global Emerging Infections Surveillance (GEIS) Section, FY2022 ProMIS ID: P0116_22_KY and FY2023 ProMIS ID P0094_23_KY.’**

5. Thank you for stating the following financial disclosure:

“Armed Forces Health Surveillance Branch (AFHSB) and its Global Emerging Infections Surveillance (GEIS) Section, FY2022 ProMIS ID: P0116_22_KY and FY2023 ProMIS ID P0094_23_KY.”

Please state what role the funders took in the study.  If the funders had no role, please state: "The funders had no role in study design, data collection and analysis, decision to publish, or preparation of the manuscript."

If this statement is not correct you must amend it as needed.

Please include this amended Role of Funder statement in your cover letter; we will change the online submission form on your behalf.

**Response 3: The funders had a role on validation of the project, administration and decision to publish**

6. When completing the data availability statement of the submission form, you indicated that you will make your data available on acceptance. We strongly recommend all authors decide on a data sharing plan before acceptance, as the process can be lengthy and hold up publication timelines. Please note that, though access restrictions are acceptable now, your entire data will need to be made freely accessible if your manuscript is accepted for publication. This policy applies to all data except where public deposition would breach compliance with the protocol approved by your research ethics board. If you are unable to adhere to our open data policy, please kindly revise your statement to explain your reasoning and we will seek the editor's input on an exemption. Please be assured that, once you have provided your new statement, the assessment of your exemption will not hold up the peer review process.

**Response 6: The authors have agreed have a data sharing plan before acceptance**

7. Please amend the manuscript submission data (via Edit Submission) to include author Wallace Bulimo, Solomon Langat, Johnson Kinyua, Nicholas Odemba, Santos Yalwala, David Oullo, Richard Ochieng, Francis Ngere, Gladys Kerich, Janet Ambale, Eunice Achieng, David Abuom, Timothy Egbo, Jaree Johnson, Elly Ojwang, John Eads, Eric Garges and Fredrick Eyase.

**Response 7: All authors have been included on the manuscript submission data**

8. Your ethics statement should only appear in the Methods section of your manuscript. If your ethics statement is written in any section besides the Methods, please move it to the Methods section and delete it from any other section. Please ensure that your ethics statement is included in your manuscript, as the ethics statement entered into the online submission form will not be published alongside your manuscript.

**Response 8: Ethics statement have been moved to methods section**

9. We note that Figure 1 in your submission contain map/satellite images which may be copyrighted. All PLOS content is published under the Creative Commons Attribution License (CC BY 4.0), which means that the manuscript, images, and Supporting Information files will be freely available online, and any third party is permitted to access, download, copy, distribute, and use these materials in any way, even commercially, with proper attribution. For these reasons, we cannot publish previously copyrighted maps or satellite images created using proprietary data, such as Google software (Google Maps, Street View, and Earth). For more information, see our copyright guidelines: <http://journals.plos.org/plosone/s/licenses-and-copyright>.

 We require you to either (a) present written permission from the copyright holder to publish these figures specifically under the CC BY 4.0 license, or (b) remove the figures from your submission:

 a. You may seek permission from the original copyright holder of Figure 1 to publish the content specifically under the CC BY 4.0 license.

 We recommend that you contact the original copyright holder with the Content Permission Form (<http://journals.plos.org/plosone/s/file?id=7c09/content-permission-form.pdf>) and the following text:

“I request permission for the open-access journal PLOS ONE to publish XXX under the Creative Commons Attribution License (CCAL) CC BY 4.0 (<http://creativecommons.org/licenses/by/4.0/>). Please be aware that this license allows unrestricted use and distribution, even commercially, by third parties. Please reply and provide explicit written permission to publish XXX under a CC BY license and complete the attached form.”

 Please upload the completed Content Permission Form or other proof of granted permissions as an "Other" file with your submission.

 In the figure caption of the copyrighted figure, please include the following text: “Reprinted from [ref] under a CC BY license, with permission from [name of publisher], original copyright [original copyright year].”

b. If you are unable to obtain permission from the original copyright holder to publish these figures under the CC BY 4.0 license or if the copyright holder’s requirements are incompatible with the CC BY 4.0 license, please either i) remove the figure or ii) supply a replacement figure that complies with the CC BY 4.0 license. Please check copyright information on all replacement figures and update the figure caption with source information. If applicable, please specify in the figure caption text when a figure is similar but not identical to the original image and is therefore for illustrative purposes only.

The following resources for replacing copyrighted map figures may be helpful:

 USGS National Map Viewer (public domain): <http://viewer.nationalmap.gov/viewer/>

The Gateway to Astronaut Photography of Earth (public domain): <http://eol.jsc.nasa.gov/sseop/clickmap/>

Maps at the CIA (public domain): <https://www.cia.gov/library/publications/the-world-factbook/index.html> and <https://www.cia.gov/library/publications/cia-maps-publications/index.html>

NASA Earth Observatory (public domain): <http://earthobservatory.nasa.gov/>

Landsat: <http://landsat.visibleearth.nasa.gov/>

USGS EROS (Earth Resources Observatory and Science (EROS) Center) (public domain): [http://eros.usgs.gov/#](http://eros.usgs.gov/)

Natural Earth (public domain): <http://www.naturalearthdata.com/>

[Note: HTML markup is below. Please do not edit.]

Reviewers' comments:

Reviewer's Responses to Questions

**Response 9: This is the caption with granted permission**. ‘**Fig 1.** **The overall map of Kenya and Kisumu County showing the site where sampling was conducted. Base maps, boundaries and shape files of Kenyan map and administrative boundaries of the County and Sub-county were derived from GADM data version 4.1 (**[**https://gadm.org**](https://gadm.org/)**) and the maps were generated using ArcGIS Version 10.2.2 (**[**http://desktop.arcgis.com/en/arcmap**](http://desktop.arcgis.com/en/arcmap)**) advanced license) courtesy of Samuel Owaka.**

**Comments to the Author**

1. Is the manuscript technically sound, and do the data support the conclusions?

The manuscript must describe a technically sound piece of scientific research with data that supports the conclusions. Experiments must have been conducted rigorously, with appropriate controls, replication, and sample sizes. The conclusions must be drawn appropriately based on the data presented.

Reviewer #1: Partly

Reviewer #2: Partly

2. Has the statistical analysis been performed appropriately and rigorously?

Reviewer #1: N/A

Reviewer #2: Yes

3. Have the authors made all data underlying the findings in their manuscript fully available?

The [PLOS Data policy](http://track.editorialmanager.com/CL0/http:%2F%2Fwww.plosone.org%2Fstatic%2Fpolicies.action%23sharing/1/010f0195cb2ffe30-355d8bf0-d2ba-4f68-a0fc-96da6cad24db-000000/CmZtyLKNAcgpAtW2fQdgq1dXZOEWNyDsmulrzUk5NEg=203) requires authors to make all data underlying the findings described in their manuscript fully available without restriction, with rare exception (please refer to the Data Availability Statement in the manuscript PDF file). The data should be provided as part of the manuscript or its supporting information, or deposited to a public repository. For example, in addition to summary statistics, the data points behind means, medians and variance measures should be available. If there are restrictions on publicly sharing data—e.g. participant privacy or use of data from a third party—those must be specified.

Reviewer #1: Yes

Reviewer #2: Yes

4. Is the manuscript presented in an intelligible fashion and written in standard English?

PLOS ONE does not copyedit accepted manuscripts, so the language in submitted articles must be clear, correct, and unambiguous. Any typographical or grammatical errors should be corrected at revision, so please note any specific errors here.

Reviewer #1: Yes

Reviewer #2: Yes

5. Review Comments to the Author

Please use the space provided to explain your answers to the questions above. You may also include additional comments for the author, including concerns about dual publication, research ethics, or publication ethics. (Please upload your review as an attachment if it exceeds 20,000 characters)

Reviewer #1: The study is very intersting, just have the time to review the different points to make your manuscript more attractive. In your study you did not use a second method to confirme the dengue virus strain and also the use of cell culture may have mask other viruses that are not so competent to be amplified in cell culture isolation which you wanted to reveal with NGS but here also the use of 550 samples is too high and could also mask the viruses which are in low quantity.

Reviewer #2: Dear Authors,
Your article is very good; however, I have few major and minor comments that could improve the quality and readability of your article.
Detail comments are attached and try to modify your manuscripts based on comments

6. PLOS authors have the option to publish the peer review history of their article ([what does this mean?](https://track.editorialmanager.com/CL0/https:%2F%2Fjournals.plos.org%2Fplosone%2Fs%2Feditorial-and-peer-review-process%23loc-peer-review-history/1/010f0195cb2ffe30-355d8bf0-d2ba-4f68-a0fc-96da6cad24db-000000/7mlRgtEr4Ob72z2u0d5mLk7l4n8L6Y2mOS8ZEyatUAQ=203)). If published, this will include your full peer review and any attached files.

If you choose “no”, your identity will remain anonymous but your review may still be made public.

**Do you want your identity to be public for this peer review?** For information about this choice, including consent withdrawal, please see our [Privacy Policy](https://track.editorialmanager.com/CL0/https:%2F%2Fwww.plos.org%2Fprivacy-policy/1/010f0195cb2ffe30-355d8bf0-d2ba-4f68-a0fc-96da6cad24db-000000/5qR7dN6X8Gbmx3yb6ExsrxRh66ylLjMVyyMPg_gjDvY=203).

Reviewer #1: No

Reviewer #2: No

[NOTE: If reviewer comments were submitted as an attachment file, they will be attached to this email and accessible via the submission site. Please log into your account, locate the manuscript record, and check for the action link "View Attachments". If this link does not appear, there are no attachment files.]

While revising your submission, please upload your figure files to the Preflight Analysis and Conversion Engine (PACE) digital diagnostic tool, [https://pacev2.apexcovantage.com/](https://track.editorialmanager.com/CL0/https:%2F%2Fpacev2.apexcovantage.com%2F/1/010f0195cb2ffe30-355d8bf0-d2ba-4f68-a0fc-96da6cad24db-000000/wAyiMJ6Ablcnocr6hbDNBKdmGgBWSkON14bl95BBCRc=203). PACE helps ensure that figures meet PLOS requirements. To use PACE, you must first register as a user. Registration is free. Then, login and navigate to the UPLOAD tab, where you will find detailed instructions on how to use the tool. If you encounter any issues or have any questions when using PACE, please email PLOS at [figures@plos.org](mailto:figures@plos.org). Please note that Supporting Information files do not need this step.

*In compliance with data protection regulations, you may request that we remove your personal registration details at any time.* [*(Remove my information/details)*](https://track.editorialmanager.com/CL0/https:%2F%2Fwww.editorialmanager.com%2Fpone%2Flogin.asp%3Fa=r/1/010f0195cb2ffe30-355d8bf0-d2ba-4f68-a0fc-96da6cad24db-000000/nfzB-BoJnPjafJRY1L8b7JspqTcoi-4wS2bPevbWL6Y=203)*. Please contact the publication office if you have any questions.*
