# Supplementary material for: Vertical transmission of Dengue virus type-3 and metagenomic virome profiles of Aedes aegypti mosquitoes collected in Kisumu, Kenya
Source: PLoS One. 2025 Jul 2;20(7):e0315492. doi: 10.1371/journal.pone.0315492 (PMC12221173; doi:10.1371/journal.pone.0315492)
Supplement: Attachment — Submitted filename: Rebuttal Letter to Reviewers.docx [file pone.0315492.s005.docx]

**Rebuttal Letter to Reviewers**

Manuscript Title**: Vertical Transmission of Dengue Virus Type-3 and Metagenomic Virome Profiles of Aedes aegypti Mosquitoes Collected in Kisumu, Kenya**

Manuscript ID**: PONE-D-24-54015R1**

Dear Editor,

We appreciate the opportunity to revise our manuscript and sincerely thank you and the reviewers for the constructive and thoughtful feedback. Below, we provide detailed responses to the comments raised, along with a summary of the corresponding changes made to the manuscript.

Reviewer

Comment:

Please review your reference list to ensure that it is complete and correct. If you have cited papers that have been retracted, please include the rationale for doing so in the manuscript text, or remove these references and replace them with relevant current references. Any changes to the reference list should be mentioned in the rebuttal letter that accompanies your revised manuscript. If you need to cite a retracted article, indicate the article’s retracted status in the References list and also include a citation and full reference for the retraction notice.

Response:

Thank you for this important observation. We have reviewed all references and confirm that they are correct, up to date, and none have been retracted. Additionally, we have revised Reference No. 41 (Line 147) to reflect a more recent and relevant citation. This has been updated in the revised manuscript.

Reviewer #2

Comment:

Dear Authors, Thank you for considering my comments. However, one important comment is not addressed. This is not only about the quality and readability of your article but also for readers. Like reference you used to classify entomological indices, different scholars will use your article and five households are enough to estimate entomological indices.

Response:

Line 203, 293 & 372: This section (Larval Indices) has been removed in Methodology, Results and discussion section.

We hope that these revisions sufficiently address the concerns raised. Thank you again for your time and consideration.

Sincerely,

Tabitha Wanjiru

Jomo Kenyatta University of Agriculture and Technology

P.O. Box 62000 – 00200

NAIROBI.

wanjirutabs@gmail.com

+254726826201

10^th^ May, 2025
